# Probiotics for glycemic and lipid profile control of the pre-diabetic patients: a randomized, double-blinded, placebo-controlled clinical trial study

**DOI:** 10.1186/s13098-023-01050-9

**Published:** 2023-04-10

**Authors:** Mina AkbariRad, Somayeh Sadat Shariatmaghani, Bibi Marjan Razavi, Hassan Mehrad Majd, Zeinab Shakhsemampour, Mehrdad Sarabi, Maryam Jafari, Setareh Azarkar, AmirAli Moodi Ghalibaf, Zahra Mazloum Khorasani

**Affiliations:** 1 Department of Internal Medicine, Faculty of Medicine, Mashhad University of Medical Sciences, Mashhad, Iran; 2 Fellowship of hematology and oncology, Department of Internal Medicine, Faculty of Medicine, Mashhad University of Medical Sciences, Mashhad, Iran; 3 Targeted Drug Delivery Research Center, Pharmaceutical Technology Institute, Mashhad University of Medical Sciences, Mashhad, Iran; 4 Department of Pharmacodynamics and Toxicology, School of Pharmacy, Mashhad University of Medical Sciences, Mashhad, Iran; 5 Clinical Research Development Unit, Ghaem Hospital, Mashhad University of Medical Sciences, Mashhad, Iran; 6 Student Research Committee, Faculty of Medicine, Mashhad University of Medical Sciences, Mashhad, Iran; 7 Student Research Committee, Anzali International Medical Campus, Guilan University of Medical Sciences, Guilan, Iran; 8 Student Research Committee, Faculty of Medicine, Birjand University of Medical Sciences, Birjand, Iran; 9 Endocrine Research Center, Mashhad University of Medical Sciences, Mashhad, Iran

**Keywords:** Pre-diabetes, Probiotic, Glucose profile, Lipid profile, Blood sugar control, Insulin resistance, Integrative medicine, Complementary medicine

## Abstract

**Background:**

Pre-diabetes is a condition in which blood glucose levels are high but not as high as in diabetic patients. However, it can lead to diabetes, making it a serious global health issue. Previous studies have shown that the gut microbiome can affect insulin sensitivity and improve glucose management, which can reduce or delay the progression of pre-diabetes to type 2 diabetes mellitus. This study was designed to investigate the effects of probiotics on glycemic and lipid profile control in pre-diabetic patients.

**Methods:**

This randomized, double-blinded clinical trial was conducted on 70 pre-diabetic patients at the Ghaem Hospital, Mashhad University of Medical Sciences, Mashhad, Iran. Participants were divided into two groups, both of which received lifestyle modification training. One of the groups also received 500 mg/day probiotic capsules for three months, while the other group received a placebo. Before and after the three-month period, systolic and diastolic blood pressure, serum insulin level, hemoglobin A1c (HbA1c), fasting blood sugar (FBS), low-density lipoprotein (LDL), high-density lipoprotein (HDL), and triglycerides (TG) were measured and compared using statistical tests to examine the effect of probiotics.

**Results:**

A total of 70 individuals participated in the trial, including 50 women (71.4%) and 20 men (28.6%), with an average age of 43.53 ± 8.54 years. At the end of the trial, the mean weight (P < 0.001), FBS (P < 0.001), HbA1c (P = 0.035), TG (P = 0.004), and LDL (P = 0.016) were significantly reduced in the intervention group, while their insulin level (P = 0.041) and HDL (P = 0.001) were significantly increased. However, mean systolic (P = 0.459) and diastolic blood pressure (P = 0.961) and insulin resistance (P = 0.235) did not show any significant difference in the intervention group from the beginning of the study.

**Conclusion:**

Our study showed that probiotic administration is effective in improving the glucose and lipid profile of pre-diabetic patients. However, it was not significantly different from the placebo.

## Introduction

Diabetes Mellitus (DM) and obesity are two major global health problems that have gained increasing attention in recent years [1]. Pre-diabetes is a complex health condition that can result from decreased insulin resistance, elevated glucose levels, and increased inflammatory cytokines [2]. Specifically, insulin resistance in the liver and peripheral tissue, as well as a decrease in sensitivity to glucose in beta cells, contribute to hyperglycemia [3]. People with pre-diabetes, like those with DM, are at risk of experiencing microvascular complications and neuropathy [4]. Type 2 diabetes accounts for 90% of all cases of diabetes and can lead to various complications, including cardiovascular disease (CVDs), renal failure, stroke, retinopathy, nervous system complications, and vascular complications, which result in significant costs to society. Type 2 diabetes mellitus and its complications significantly impact quality of life. Therefore, preventing diabetes mellitus through screening, lifestyle interventions, and food supplements is crucial, particularly for high-risk individuals [5, 6]. According to the Centers for Disease Control and Prevention (CDC), approximately 84.1 million American adults, or one out of three, had pre-diabetes in 2017. In 2019, the International Diabetes Federation reported that 7.5% of the adult population, approximately 373.9 million people aged 20 to 79, had impaired glucose tolerance, with an estimated 548.4 million people projected to have the condition by 2045 [7].

Previous studies have indicated that early intervention, particularly during the pre-diabetes stage, can prevent type 2 DM [8]. Elevated endotoxin concentration in adults is associated with insulin resistance and CVDs and increases the risk of developing type 2 DM. Approximately 9.3% to 55% of people with pre-diabetes develop type 2 DM within three years [9]. Therefore, effective, timely, and cost-effective interventions are crucial to preventing the progression of this chronic disease [10].

Recently, gut microbiota has been considered a new treatment for metabolic diseases such as diabetes [8, 11]. Gut microbiota can play an important role in epithelial homeostasis, the synthesis and oxidation of fatty acids, the host immune system, and can also influence host nutrition and energy. Certain probiotic species have been shown to improve inflammatory markers, insulin sensitivity, and lipid profiles in type 2 DM [12, 13].

Previous animal model studies have demonstrated that probiotics have positive effects on insulin sensitivity and beta-cell dysfunction by regulating signaling pathways [14, 15]. Clinical trials investigating the role of microbiota in pre-diabetes and obesity have shown that the effect of probiotics on metabolic variables such as weight, body mass index (BMI), glucose, postprandial insulin, and HbA1c can differ. Similarly, lipid profile variables have been associated with reductions in total cholesterol, LDL, and triglycerides, while some studies report no difference in lipid profile [16].

Therefore, no consensus currently exists regarding probiotics’ effect on pre-diabetes control or treatment. In this regard, the present study is designed to investigate the effects of probiotics on glucose and lipid profile control in pre-diabetic patients.

## Methods

### Study design

The present double-blinded, randomized, parallel-controlled clinical trial was conducted to determine the effectiveness of probiotics in pre-diabetes treatment. Participants were randomly assigned to take probiotics or placebo capsules using a convenience sampling method. The protocol for conducting the present study was approved by the ethics committee of Mashhad University of Medical Sciences, Mashhad, Iran (IR.MUMS.fm.REC. 1396.451), and registered with the Iranian Registry of Clinical Trials (IRCTID: IRCT20180527039866N1). The study was conducted in compliance with the principles of confidentiality and privacy. Collection and analysis of patient information were performed anonymously and using a code to prevent disclosure. Participants were free to leave the study at any time without providing any explanation or reason.

### Participants

We enrolled 70 pre-diabetic patients, whose disease diagnosis had been confirmed by an internist or endocrinologist according to laboratory criteria, aged between 30 and 65 years old, recruited from patients referred to the specialized internal clinic of Ghaem Hospital, Mashhad University of Medical Sciences, Mashhad, Iran, and who provided informed consent to participate in the project. Eligible individuals were those with fasting plasma glucose concentrations of 100–125 mg/dL, 2-h glucose tolerance test levels of 140–199 mg/dL, or hemoglobin A1C between 5.7%-6.4% [17]. Participants should have controlled glycemic and lipid profile levels. Exclusion criteria were defined as a previous diagnosis of diabetes or pre-diabetes, a glomerular filtration rate less than 60%, a history of known gastrointestinal malabsorption or chronic diarrhea, receipt of probiotics, antibiotics, aspirin, or group B vitamins during the last three months, recent medication with pioglitazone, dissatisfaction to continue participating in the project, failure to return for visits and follow-up according to the communicated schedule.

The allocation of patients to the mentioned groups was done using sealed envelopes. After the sealed envelopes were randomly distributed among the patients, a person outside the main research team opened the envelopes and prescribed the appropriate medication to the patient based on the letter inside the envelope, and provided necessary explanations. The examining doctors and the patients themselves were not aware of the nature of the treatment received, and blinding was done in a 2-way manner.

### Interventions

The intervention carried out involved a change in the treatment method for pre-diabetic patients. All patients were trained in lifestyle modification. However, in addition to lifestyle modification, one group took probiotic capsules with a dose of 500 mg/day, and another group was treated with placebo capsules [18].

The participants were randomly divided into two groups using a table of random numbers taken from an internet website (https://www.randomization.com/). The first group received a placebo, and the second group received 500 mg probiotic capsules "Lactocare®" [ZistTakhmir, Iran], containing seven strains (including Lactobacillus casei, Lactobacillus acidophilus, Lactobacillus rhamnosus, Lactobacillus bulgaricus, Bifidobacterium breve, Bifidobacterium longum, Streptococcus thermophiles with prebiotic fructooligosaccharide).

Lifestyle modification lessons, such as 150 min of weekly exercise and nutrition modification (e.g. limiting portion sizes of refined carbohydrate foods such as white bread, white rice, and white pasta, incorporating fiber to reach a goal of 25 to 30 g per day by eating a variety of fruits, vegetables, and whole grains, limiting saturated and trans fats by choosing lean protein and low-fat dairy), were taught to all patients in both groups. Additionally, all the patients in both groups were advised to refrain from consuming milk, yogurt, and other probiotics-enriched dairy products during the study period.

### Outcomes

All patients were visited and examined once at the beginning of the study and after three months. Initially, patients were referred to the laboratory to measure serum levels of insulin, Fasting Blood Sugar (FBS), LDL, HDL, triglyceride (TG), HbA1c, and Homeostatic Model Assessment for Insulin Resistance (HOMA-IR). On the second visit (3 months after the first visit), the clinical information of blood pressure, weight, height, and body mass index and the para-clinical information of Insulin, FBS, LDL, HDL, TG, HbA1c were recorded in a checklist. During the three months of the study, the patients were followed up by phone calls, and their questions were answered. At the end of the study, the completed checklists collected during the mentioned visits were classified according to patient information.

### Statistical analysis

After collecting and categorizing the data, it was entered into the software. Statistical analysis was done using Statistical Package for Social Sciences (SPSS), version 26. Descriptive statistics, including central indicators, dispersion, and frequency distribution, were reported. The relationship between distinct qualitative variables was measured using Chi-square and Fisher's exact statistical tests. The normality or non-normality of quantitative data distribution was also measured using the Kolmogorov–Smirnov statistical test. Where normal distribution and variances were equal, an independent t-test was applied to compare quantitative variables between two groups. In cases where the distribution was not normal or the condition of equality of variances did not exist, the Mann–Whitney statistical test was conducted. To compare quantitative variables within each group (at the beginning and end of the study), paired t-test was used in cases where the distribution was normal, and the Wilcoxon test was used in the remaining cases. Results with P < 0.05 were considered to be statistically significant.

## Result

**Fig. 1:**
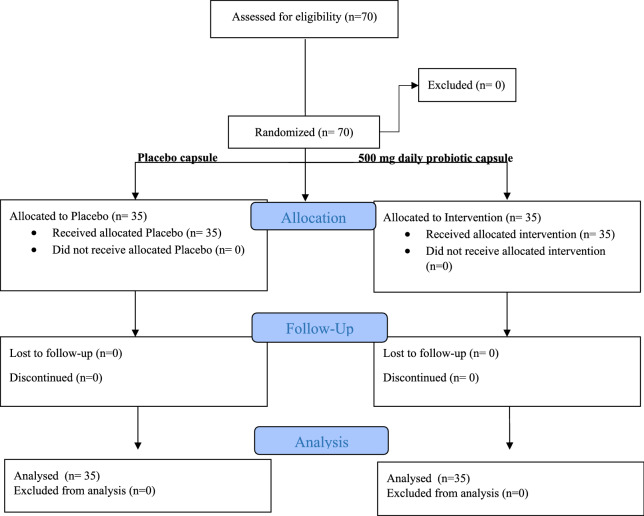
The CONSORT diagram of the present study

In total, 70 individuals participated in this randomized controlled trial, comprising 50 women (71.4%) and 20 men (28.6%), with an average age of 43.53 ± 8.54 years. The participants were divided into two groups: an intervention group receiving probiotics and a placebo group, with 35 individuals in each group (Fig. 1).

The general characteristics of the study participants are summarized in Table 1. There were no significant differences in the mean age (P = 0.630), weight (P = 0.417), and body mass index (BMI) (P = 0.557) between the intervention and placebo groups, indicating that the two groups were well-matched in terms of these variables.

**Table 1:** Characteristics of studied patients in two groups

Variable	Group Mean ± SD		P-value
	Placebo	intervention	
Age(year)	43.06 ± 8.53	44 ± 8.66	0.630^a^
weight(kg)	85.40 ± 16.59	81.94 ± 16.81	0.417^b^
BMI(kg/${m}^{2}$)	31.41 ± 3.97	30.74 ± 3.56	0.557^a^

Gender	Group N (%)		P-value
	Placebo	intervention	
Female	24(68.6%)	26(74.3%)	0.597^b^
Male	11(31.4%)	9(25.7%)	

^a^Mann-Whitney test

^b^Chi-square test

The mean variables of SBP (P = 0.787), DBP (P = 0.755), FBS (P = 0.897), HbA1c (P = 0.595), insulin (P = 0.601), HOMA-IR (P = 0.557), and lipid profile parameters including TG (P = 0.773), LDL (P = 0.724), and HDL (P = 0.846) between patients in the intervention and placebo groups at the beginning of the study were calculated. The results of this comparison showed that at the beginning of the study, the mean of none of the mentioned variables was significantly different between the two groups. Calculating the mean of the mentioned variables in the intervention and placebo groups at the end of the trial and comparing the resulting values showed that at the end of the study, the mean of these variables in the two groups was not significantly different. The results of this comparison are listed in Table 2.

**Table 2:** Mean and Standard Deviation (SD) of the Characteristics of the participants at the initial and the end of the intervention, the variation of the variables during the intervention, and comparison of the parameters between the groups after the intervention

Variable	Group (Before Intervention) Mean ± SD		P-value	Group (After Intervention) Mean ± SD		P-value	Variations		P-value	P-value (comparison of the groups after intervention)	
	Placebo	Intervention		Placebo	Intervention		Placebo	Intervention		Placebo	Intervention
Weight (kg)	85.40 ± 16.59	81.94 ± 16.81	0.417^b^	82.69 ± 16.42	79.34 ± 16.81	0.503^b^	− 2.71 ± 2.33	− 2.60 ± 2.56	0.774^b^	< 0.001^d^	< 0.001^d^
SBP (mmHg)	123.86 ± 10.65	125.29 ± 12.48	0.787^b^	124.71 ± 7.76	126.86 ± 6.68	0.251^b^	+ 0.86 ± 11.08	+ 1.57 ± 12.41	0.674^b^	0.755^d^	0.459^c^
DBP (mmHg)	80.71 ± 7.39	81.57 ± 8.38	0.755^b^	81.11 ± 7.55	82 ± 11.13	0.990^b^	+ 0.40 ± 10.25	+ 2.71 ± 2.33	0.901^b^	0.819^d^	0.961^d^
FBS (mg/dL)	109.17 ± 6.90	108.73 ± 6.72	0.897^b^	95.11 ± 10.55	96.17 ± 9.39	0.495^b^	− 14.06 ± 10.18	-12.66 ± 10.42	0.764^b^	< 0.001^d^	< 0.001^d^
HbA1C (%)	5.69 ± 0.88	5.58 ± 0.65	0.595^b^	5.48 ± 0.85	5.28 ± 0.78	0.484^b^	− 0.21 ± 0.82	− 0.29 ± 0.79	0.972^a^	0.133^c^	0.035^c^
Insulin level (µIU/mL)	15.73 ± 11.03	13.67 ± 9.30	0.601^b^	22.14 ± 26.20	22.35 ± 25.82	0.796^a^	+ 6.41 ± 30.68	+ 8.68 ± 25.78	0.470^b^	0.451^d^	0.041^d^
HOMA-IR	4.25 ± 3	3.64 ± 2.41	0.557^b^	5.18 ± 6.24	5.34 ± 6.20	0.747^b^	+ 0.92 ± 7.54	+ 1.71 ± 6.18	0.347^b^	> 0.999^d^	0.235^d^
TG (mg/dL)	146.31 ± 50.54	147.94 ± 40.82	0.773^a^	117.40 ± 42.23	132.89 ± 83.69	0.597^b^	− 28.91 ± 55	− 15.06 ± 95.71	0.972^b^	0.004^c^	0.004^c^
LDL(mg/dL)	121.09 ± 37.71	117.29 ± 37.28	0.724^a^	104.74 ± 30.14	102.49 ± 29.80	0.906^b^	− 16.34 ± 34.51	− 14.80 ± 34.55	0.823^b^	0.008^c^	0.016^c^
HDL(mg/dL)	45.54 ± 8.54	44.66 ± 6.49	0.846^b^	49.26 ± 7.58	48.54 ± 6.63	0.846^b^	+ 3.71 ± 7.39	+ 3.89 ± 7.08	0.948^b^	0.005^d^	0.001^d^

^a^independent T-test /

^b^Mann-Whitney test /

^c^paired t-test /

^d^Wilcoxon statistical test

In the next step, the variations in each of the investigated variables were calculated separately in the intervention and placebo groups. The results of this comparison indicated that there were no significant differences in the absolute value of the mean change in any of the investigated variables between the two groups (Tables 2, 3). Specifically, between-groups differences p-values were as follows: weight (P = 0.774), SBP (P = 0.674), DBP (P = 0.901), FBS (P = 0.764), HbA1c (P = 0.972), insulin level (P = 0.470), HOMA-IR (P = 0.347), TG (P = 0.972), LDL (P = 0.823), and HDL (P = 0.948) (Table 3).

**Table 3:** Mean, Inter Quartile Range (IQR), and 95% Confidence Interval (95%CI) of the Characteristics of the participants at the initial and the end of the intervention, the variation of the variables during the intervention, and comparison of the parameters between the groups after the intervention

Variable	Group (Before Intervention) Median (IQR)		P-value	Group (After Intervention) Median (IQR)		P-value	Between groups variations Mean difference 95% CI	P-value	P-value (comparison of the groups after intervention)	
	Placebo	Intervention		Placebo	Intervention				Placebo	Intervention
Weight (kg)	85.0 (69–100)	74.0 (69–92)	0.417^b^	80 (68–98)	72 (68–90)	0.503^b^	− 3.35 (− 4.58, − 11.28)	0.774^b^	< 0.001^d^	< 0.001^d^
SBP (mmHg)	120 (120–130)	120 (120–130)	0.787^b^	125 (120–130)	125 (125–130)	0.251^b^	2.15 (− 1.30, − 5.60)	0.674^b^	0.755^d^	0.459^c^
DBP (mmHg)	80 (75–85)	80 (75–90)	0.755^b^	80 (75–85)	80 (75–85)	0.990^b^	0.89 (− 3.65, − 5.43)	0.901^b^	0.819^d^	0.961^d^
FBS (mg/dL)	107 (104–115)	107 (104–112)	0.897^b^	95 (88–100)	96 (89–102)	0.495^b^	1.06 (− 3.70, − 5.82)	0.764^b^	< 0.001^d^	< 0.001^d^
HbA1C (%)	5.80 (4.8–6.1)	5.80 (5.0–5.9)	0.595^b^	5.4 (4.8–5.9)	5.4 (4.7–5.8)	0.484^b^	− 0.2 (− 0.19, 0.59)	0.972^*^	0.133^c^	0.035^c^
Insulin level (µIU/mL)	12.2 (6.8–27.1)	11 (7.6–18.3)	0.601^b^	15.5 (6–25)	15.5 (8.1–25)	0.796^a^	0.21 (− 12.20, 12.62)	0.470^b^	0.451^d^	0.041^d^
HOMA-IR	3.24 (1.91–7.30)	3.15 (1.93–4.88)	0.557^b^	3.86 (1.51–5.98)	3.86 (1.82–5.98)	0.747^b^	0.16 (− 2.81, 3.13)	0.347^b^	> 0.999^d^	0.235^d^
TG(mg/dL)	144 (105–173)	148 (130–173)	0.773^a^	110 (84–141)	117 (91–144)	0.597^b^	15.49 (− 16.13, 47.11)	0.972^b^	0.004^c^	0.004^c^
LDL(mg/dL)	114 (91–147)	112 (91–145)	0.724^a^	99 (84–128)	96 (84–128)	0.906^b^	-2.25 (− 12.05, 16.55)	0.823^b^	0.008^c^	0.016^c^
HDL(mg/dL)	46 (40–48)	46 (39–49)	0.846^b^	50 (43–54)	49 (42–55)	0.846^b^	0.72 (− 2.68, 4.12)	0.948^b^	0.005^d^	0.001^d^

^a^independent T-test

^b^Mann-Whitney test

^c^paired t-test

^d^Wilcoxon statistical test

In the last step, the variations in the studied variables from the initiation to the end of the study were evaluated separately for each group. In the placebo group, there was a significant reduction in weight (P < 0.001), FBS (P < 0.001), TG (P = 0.004), and LDL (P = 0.008) of the patients at the end of the trial compared to the beginning of the study. Additionally, there was a significant increase in the level of HDL (P = 0.005). However, SBP (P = 0.755), DBP (P = 0.819), HbA1c (P = 0.133), insulin level (P = 0.451), and HOMA-IR (P = 0.347) of the patients at the beginning and end of the trial were not significantly different. In the intervention group, SBP (P = 0.459), DBP (P = 0.961), and HOMA-IR (P = 0.235) of the patients at the end of the trial were not significantly different from the beginning of the study. However, the average weight (P < 0.001), FBS (P < 0.001), HbA1c (P = 0.035), TG (P = 0.004), and LDL (P = 0.016) of the patients decreased at the end of the trial, and the level of insulin (P = 0.041) and HDL (P = 0.001) of the patients also significantly increased. The results of these comparisons are summarized in Table 2.

## Discussion

The present clinical trial investigated the effects of probiotics on the glucose and lipid profiles of pre-diabetic patients. Our comparisons revealed that none of the variables, including FBS, LDL, HDL, TG, and HbA1c, were significantly different between the placebo and intervention groups at the beginning or end of the study. Notably, there was a significant difference in the absolute value of the mean change. There were no differences between the two groups in any of the studied variables. In the placebo group, the mean weight, FBS, TG, and LDL of the patients decreased significantly, compared to the beginning of the study, and there was a significant increase in the HDL levels of the patients. However, the mean of other variables did not change significantly. In the intervention group, the mean weight, FBS, HbA1c, TG, and LDL of the patients decreased at the end of the trial, and the mean serum insulin levels and HDL of the patients increased significantly. However, the SBP and DBP of the patients and their HOMA-IR at the end of the trial did not show a significant difference from the beginning of the study.

A double-blind clinical trial showed that administering probiotics for eight weeks to pre-diabetic patients significantly reduced HbA1C, but no significant differences were observed in FBS, LDL, HDL, and TG. Despite our study, this trial was conducted over a shorter period of time and with a different type of Lactobacillus. It is notable that in this study, the diet of patients also included fermented milk products [19].

Recently, a systematic review study by Salles et al. investigated the effects of prescribing probiotics on reducing insulin resistance in human and animal model studies. The findings of 27 animal model studies indicated the significant effectiveness of probiotics in reducing insulin resistance, improving lipid profiles, and reducing inflammatory factors in animals. Moreover, most of the reviewed clinical trial studies suggested that the consumption of probiotics was associated with improvement in indices related to insulin resistance; however, two studies did not report such an effect. Although administering probiotics can be useful in improving insulin resistance, more studies are necessary due to the heterogeneity of the trials conducted in this field [20]. As mentioned, in our study, administering probiotics did not have a significant effect on reducing insulin resistance. Of course, it should be mentioned again that we assessed only pre-diabetic patients in our study, which may partially justify the observed differences.

In another double-blinded clinical trial study conducted by Kasaian et al., 120 prediabetic adult patients were divided into three groups including placebos, probiotics, and synbiotics, and then evaluated for the effects of each compound over 6 months. The results of the study showed that probiotic supplements significantly altered the microbiota of the gastrointestinal tract, and taking probiotics can be considered as a preventive or treatment method for obesity and diabetes [21]. Moreover, a previous study revealed that the consumption of probiotics for 24 weeks caused a significant decrease in HbA1c compared to the placebo, but there was no significant difference in reducing insulin resistance [22]. In our study, the consumption of probiotics led to a significant decrease in HbA1c. However, in contrast to the study by Kasaian et al., the observed difference was not significantly different from the placebo in ours. As the duration of using probiotics in our study was 12 weeks, we did not examine synbiotics or stool samples of the patients, and we did not evaluate the results in terms of microbiota changes, so the results could be varied.

A systematic review study by Xian Wang et al. indicated that probiotic supplements reduced the values of HbA1c and prevented the increase of total cholesterol, while its effect on lowering cholesterol levels is yet to be confirmed. However, they concluded that the consumption of probiotics and synbiotic compounds is more effective than probiotics alone [23]. In our study, we only investigated the effect of probiotics and did not study their effectiveness with other compounds. Also, we did not measure the total cholesterol level of the patients, but our trial revealed that taking probiotic supplements is associated with a significant decrease in HbA1c.

It is important to mention the role of Complementary and Alternative Medicine (CAM) in chronic disease control and management. In fact, CAM is known as therapies and practices that are not considered a part of modern medicine [24–26]. Despite the different types of CAMs presented at the current time, Mind–Body Medicine, Manipulative and Body-Based Practices, Energy Medicine, Whole medical systems, and Biologically-Based Practices are the top five domains of CAM [27]. Previous studies have revealed some aspects of CAMs not only in management but also in the treatment of chronic diseases. Interestingly, a study by Hashempur et al. demonstrated a high prevalence of medicinal plants’ use among patients with dyslipidemia, which was associated with the duration of dyslipidemia, patients’ viewpoints about herbal preparations’ synergistic positive effects, and their fewer side effects). Moreover, Alyasin et al. revealed that oral supplementation of whey protein could improve the symptoms of contact dermatitis compared with a placebo [28]. However, the complementary use of alkaloid berberine capsule with a dose of 500 mg per day did not show better outcomes compared with the placebo in patients with schizophrenia in a study by Sarani et al. [29].

Generally, despite a large number of studies and trials conducted on the effect of probiotics on the control of weight, glucose profile, lipid profile, and other metabolic indicators in different populations, the results of these studies are different and influenced by the diversity and heterogeneity of the investigated societies and diverse methodologies. It is not easy to give a definite opinion about the effectiveness of these compounds.

It should be noted that in our study, in addition to the improvements observed in the intervention group, the mean weight, FBS, and lipid profile parameters (TG, LDL, and HDL) of the patients in the placebo group were significantly improved at the end of the trial. To justify this finding, it should be considered that all the patients in both groups were given recommendations related to diet and increasing physical activity at the initiation of the study. The improvement in the placebo group's parameters could probably be related to the following physical activity and nutritional recommendations.

The present study was one of the few studies on Iranian pre-diabetic patients that investigated the effectiveness of probiotics on their glucose and lipid profiles. Moreover, the sufficient sample size of the present study, which was conducted in one of the largest diabetes centers in the northeast of Iran, could be another strong point of this study. However, there are several limitations to this study. Unlike some previous studies in this field, we have only evaluated the effectiveness of probiotics. This makes us suggest further studies to implement other alternative and complementary medications for future trials. Additionally, we studied the effect of probiotics on glucose and lipid profile parameters in a short period of time (12 weeks). Finally, it should be considered that in our study, we investigated the addition of probiotics in the form of capsules and a single dose to routine medications.

## Conclusion

The current study indicated that the daily use of probiotics in pre-diabetic patients after three months leads to weight loss and improvements in both glucose and lipid profile parameters. However, its effectiveness is not significantly different from the placebo group. Moreover, the consumption of probiotics did not have a significant effect on the patient's blood pressure and insulin resistance. Considering the limited trials in our country, Iran, and the inconsistent and sometimes contradictory results of studies conducted in other countries, it is necessary to conduct more studies to illuminate the effect of probiotics on glycemic and lipid profile parameters.

## Data Availability

The data that support the findings of this study are available on request from the corresponding author. The data are not publicly available due to privacy or ethical restrictions.

## Abbreviations

DM: Diabetes mellitus
CVDs: Cardiovascular diseases
CDC: Centers for Disease Control and Prevention
LDL: Low density lipoporetein
HbA1c: Hemoglobin A1C
HDL: High density lipoprotein
HOMA-IR: Homeostatic model assessment for insulin resistance
TG: Triglyscride
FBS: Fasting blood sugar
SPSS: Statistical Package for Social Sciences
CAM: Complementary and alternative medicine
